# Reference values for maximum oxygen uptake relative to body mass in Dutch/Flemish subjects aged 6–65 years: the LowLands Fitness Registry

**DOI:** 10.1007/s00421-021-04596-6

**Published:** 2021-02-01

**Authors:** Geertje E. van der Steeg, Tim Takken

**Affiliations:** 1 Faculty of Medicine, Utrecht University, UMC Utrecht, Utrecht, The Netherlands; 2 Child Development & Exercise, Wilhelmina Children’s Hospital, University Medical Center Utrecht, Room KB02.056.0, PO Box 85090, NL 3508 AB Utrecht, The Netherlands; 3 The Physiology Academy, Alphen aan den Rijn, The Netherlands

**Keywords:** Reference values, Oxygen consumption, Cardiorespiratory fitness, Exercise test, Ergometry

## Abstract

**Background:**

The maximum oxygen uptake (VO_2_max) during cardiopulmonary exercise testing (CPET) is considered the best measure of cardiorespiratory fitness.

**Aim:**

To provide up-to-date reference values for the VO_2_max per kilogram of body mass (VO_2_max/kg) obtained by CPET in the Netherlands and Flanders.

**Methods:**

The Lowlands Fitness Registry contains data from health checks among different professions and was used for this study. Data from 4612 apparently healthy subjects, 3671 males and 941 females, who performed maximum effort during cycle ergometry were analysed. Reference values for the VO_2_max/kg and corresponding centile curves were created according to the LMS method.

**Results:**

Age had a negative significant effect (p < .001) and males had higher values of VO_2_max/kg with an overall difference of 18.0% compared to females.

Formulas for reference values were developed:

- Males: VO_2_max/kg = − 0.0049 × age^2^ + 0.0884 × age + 48.263 (*R*^2^ = 0.9859; SEE = 1.4364)
- Females: VO_2_max/kg = − 0.0021 × age^2^ − 0.1407 × age + 43.066 (*R*^2^ = 0.9989; SEE = 0.5775).

Cross-validation showed no relevant statistical mean difference between measured and predicted values for males and a small but significant mean difference for females. We found remarkable higher VO_2_max/kg values compared to previously published studies.

**Conclusions:**

This is the first study to provide reference values for the VO_2_max/kg based on a Dutch/Flemish cohort. Our reference values can be used for a more accurate interpretation of the VO_2_max in the West-European population.

## Introduction

Cardiorespiratory fitness (CRF) has been extensively studied in recent years, as the increase in cardiovascular diseases (CVD) is an expanding problem worldwide (Laxmi et al. 2014). Low levels of CRF have been identified as a potential risk factor for CVD and all-cause mortality; moreover, substantial health benefits might be gained by improvements in CRF, which can be obtained by physical activity and exercise (Rapp et al. 2018; Ross et al. 2016). Therefore, CRF is now identified as an important marker of cardiovascular health and has even been recommended as a new vital sign by the American Heart Association (Ross et al. 2016). Cardiopulmonary Exercise Testing (CPET) allows assessment of CRF. The measurement of the maximum amount of oxygen uptake during exercise, referred to as the VO_2_max, is in particular relevant in evaluating an individual’s aerobic fitness (Mezzani et al. 2009; Rapp et al. 2018; Takken et al. 2019) and is considered the best measure of CRF and exercise capacity (Fletcher et al. 2013).

In physiological terms, the VO_2_max is the maximum level of oxygen uptake that can be consumed during exhausting exercise with large muscle groups (Fletcher et al. 2013; Herdy and Uhlendorf 2011; Mezzani et al. 2009). As a result, the VO_2_ level reaches a plateau phase. For this to happen, achievement of truly maximal effort is essential (Mezzani et al. 2009). An important component of the value of VO_2_max, that is up to 50%, is being established by hereditary capacity. However, there are several other determinants influencing the level of VO_2_max, in particular age and sex, although body size, exercise training habits, lifestyle, and cardiovascular status play a role, as well (Almeida et al. 2014; Fletcher et al. 2013; Guazzi et al. 2012, 2018; Herdy and Caixeta 2016; Kaminsky et al. 2013; Koch et al. 2009; Mezzani et al. 2009; Ross et al. 2016; Takken et al. 2019). For instance, it has been observed that levels of VO_2_max reach maximum values between the age of 15 and 30, and decrease progressively after that age. Furthermore, it has been found that males have higher levels of VO_2_max compared to females, supposedly because of differences in muscle mass, haemoglobin levels, and cardiac stroke volume (Dubowy et al. 2008; Fletcher et al. 2013; Guazzi et al. 2012; Kaminsky et al. 2013; Koch et al. 2009; Mezzani et al. 2009; Takken et al. 2019).

Determining the level of VO_2_max can be beneficial in different settings. For example, it can be used to assess the response to exercise training. Besides, the VO_2_max is convenient for evaluating CRF in patients with for instance heart or lung diseases as well as gauging their therapeutic efficacy and has been consistently determined a prognostic marker for pre-surgical risk. Furthermore, training intensity and training targets may be established by using percentages of VO_2_max, which can be useful for healthy individuals and athletes, as well (Fletcher et al. 2013; Guazzi et al. 2018).

The VO_2_max can be directly measured by incremental exercise using respiratory gas analysis, which is considered the golden standard (Almeida et al. 2014; Fletcher et al. 2013; Rapp et al. 2018; Ross et al. 2016; Takken et al. 2019). For the interpretation of a person’s VO_2_max, reliable reference values are extremely important. Due to the close correlation existing between the VO_2_max and both age and sex, it is critical to interpret these values using age- and gender-specific reference values (Rapp et al. 2018).

Worldwide frequently used age- and gender-specific reference values are proposed by Jones et al. in 1985 and Wasserman et al. in 2005. However, these reference values were obtained years ago and were based on, respectively, Canadian and American cohorts. Nevertheless, in the Netherlands and other European countries, these reference values are still widely used, but in clinical practice, these are considered quite low, especially for young adults. Hence, up-to-date and dependable reference values based on a Dutch cohort are highly needed. Furthermore, most research in this area has focused on specific age groups, for instance children, elderly, or adults in general. Only few studies have provided reference values for a broader range of ages (Takken et al. 2019).

The aim of this study is to analyse the interaction between the VO_2_max per kilogram (VO_2_max/kg) and both age and sex and develop reference values using these two determinants, based on the Dutch population. For this purpose, we applied a large apparently healthy Dutch/Flemish cohort with a broad age range, including children from the age of 6 years to adults of 65 years.

## Methods

### Study design

This study was carried out using existing data from the LowLands Fitness Registry. This database contains exercise testing data from 11 centres in the Netherlands and Belgium. These centres include Diving Medical Center Den Helder, Erasmus Medical Center Rotterdam, Hospital Jan Portaels Vilvoorde, In2Motion Sports Bureau, InspanningLoont Centre Utrecht, Isala Hospital Zwolle, Maxima Medical Center Veldhoven, Ministry of Defense Testing Center Soesterberg, Radboud UMC Nijmegen, St Anna Hospital Geldrop, and University of Applied Sciences Utrecht. These centres submitted de-identified exercise testing data to the LowLands Fitness Registry. The Medical Ethics Committee of the UMC Utrecht in the Netherlands has approved the study (protocol 16/167) (Van de Poppe et al. 2019).

### Subjects

For this study, we used data from 4637 subjects from the Lowlands Fitness Registry of which known athletes, smokers, and subjects with a Body Mass Index (BMI) > 30 were already excluded. From these subjects, we excluded the participants who did not perform a maximum effort, as well. A maximum effort was determined as a respiratory exchange ratio (RER) of ≥ 1.0 (Kokkinos et al. 2018) and a minimum of 85% of the predicted maximum heart rate. We implemented Tanaka’s equation for the prediction of maximum heart rate: [208 – (0.7 × age)] (Tanaka et al. 2001). Relevant subject characteristics and data distribution of the study participants are listed, respectively, in Tables 1 and 2.

**Table 1:** Subject characteristics of the study population^a^

Characteristics	Age categories (years)							
	5–9 (*n* = 40 males, 31 females)	10–14 (*n* = 134 males, 96 females)	15–19 (*n* = 271 males, 74 females)	20–29 (*n* = 1127 males, 244 females)	30–39 (*n* = 934 males, 246 females)	40–49 (*n* = 809 males, 164 females)	50–59 (*n* = 324 males, 76 females)	≥ 60 (*n* = 32 males, 10 females)
*Males*								
Age (years)	8.83 ± 0.93	12.41 ± 1.41	18.33 ± 1.43	25.60 ± 2.79	35.01 ± 2.89	45.16 ± 2.77	53.94 ± 2.43	62.52 ± 1.37
Weight (kg)	32.2 ± 6.0	45.3 ± 9.8	73.5 ± 11.5	80.9 ± 9.1	84.2 ± 9.6	85.3 ± 9.3	84.2 ± 8.9	81.4 ± 9.5
Height (cm)	138 ± 7	158 ± 12	181 ± 7	183 ± 7	183 ± 7	183 ± 6	182 ± 6	180 ± 5
BMI (kg/m^2^)	16.9 ± 2.1	17.9 ± 2.3	22.4 ± 2.9	24.2 ± 2.2	25.1 ± 2.3	25.5 ± 2.3	25.5 ± 2.2	25.0 ± 2.3
*Females*								
Age (years)	8.83 ± 0.71	12.43 ± 1.50	17.57 ± 1.61	25.24 ± 2.68	35.30 ± 2.93	44.54 ± 2.76	54.22 ± 2.78	61.96 ± 1.68
Weight (kg)	32.7 ± 5.0	48.1 ± 11.6	62.4 ± 7.3	66.6 ± 7.7	66.8 ± 8.9	69.0 ± 8.3	68.2 ± 8.7	69.5 ± 5.3
Height (cm)	139 ± 8	159 ± 10	171 ± 6	171 ± 6	170 ± 6	170 ± 6	168 ± 6	168 ± 6
BMI (kg/m^2^)	17.0 ± 1.8	18.8 ± 3.2	21.4 ± 2.2	22.7 ± 2.3	23.0 ± 2.6	23.8 ± 2.5	24.3 ± 2.6	24.8 ± 1.9

^a^Data are presented as mean ± SD

**Table 2:** Data distribution in males and females^a^

Data distribution	Age categories (years)							
	5–9 (*n* = 40 males, 31 females)	10–14 (*n* = 134 males, 96 females)	15–19 (*n* = 271 males, 74 females)	20–29 (*n* = 1127 males, 244 females)	30–39 (*n* = 934 males, 246 females)	40–49 (*n* = 809 males, 164 females)	50–59 (*n* = 324 males, 76 females)	≥ 60 (*n* = 32 males, 10 females)
*Males*								
VO_2_max (ml min^−1^ kg^−1^)	45.86 ± 5.94	49.65 ± 8.76	50.63 ± 7.97	47.67 ± 6.49	45.50 ± 7.62	42.61 ± 8.40	38.50 ± 8.97	38.54 ± 8.90
HRmax (bpm)	187 ± 10	190 ± 8	193 ± 10	189 ± 10	183 ± 10	175 ± 11	169 ± 12	162 ± 11
RER (VCO_2_/VO_2_)	1.12 ± 0.07	1.13 ± 0.07	1.16 ± 0.08	1.18 ± 0.08	1.18 ± 0.08	1.14 ± 0.08	1.13 ± 0.08	1.16 ± 0.08
*Females*								
VO_2_max (ml min^−1^ kg^−1^)	41.48 ± 5.83	42.00 ± 6.46	40.53 ± 6.29	39.51 ± 8.76	35.84 ± 8.03	34.19 ± 8.66	31.00 ± 8.81	29.45 ± 7.83
HRmax (bpm)	189 ± 8	192 ± 9	190 ± 8	183 ± 10	176 ± 10	171 ± 10	167 ± 12	159 ± 9
RER (VCO_2_/VO_2_)	1.11 ± 0.07	1.15 ± 0.07	1.15 ± 0.09	1.13 ± 0.09	1.13 ± 0.09	1.14 ± 0.08	1.16 ± 0.09	1.17 ± 0.08

^a^Data are presented as mean ± SD

### Testing protocol

All exercise tests were performed using electromagnetically braked cycle ergometers. These ergometers came from distinctive manufacturers, that is Lode BV, Groningen, the Netherlands and Ergoline, Bitz, Germany (Van de Poppe et al. 2019). The VO_2_max was defined as the maximum amount of oxygen uptake during maximum effort measured around sea level and was expressed in ml min^−1^ kg^−1^. This value was measured with a calibrated respiratory gas-analysis system. These systems were also from distinctive manufacturers, in particular from Cortex Metalyzer, Leipzig, Germany; Carefusion, Hoghberg, Germany; Geratherm, Bad Kissingen, Germany; Cosmed, Rome, Italy; and Medisoft, Sorrines, Belgium. The equipment was calibrated before every exercise test in accordance with the manufacturer’s instruction (Van de Poppe et al. 2019).

### Statistical analysis

Statistical analysis was performed using the SPSS Statistics software, version 25 (IBM Corp. Released 2017. IBM SPSS statistics for Windows, Version 25.0. Armonk, NY: IBM Corp.). Our data were modelled to create reference values using LMS Chartmaker Pro program™, version 2.54. This program works according to the LMS method, which is a way to summarize the distribution of the data as it changes via three curves representing the Lambda (L: the Box-Cox power describing the skewness), Median (M), and the generalised coefficient of variation (S). With a penalised likelihood function, the three curves were fitted as cubic splines using non-linear regression (Vamvakas et al. 2019; Van de Poppe et al. 2019). Hence, centile curves were created, representing the percentiles of P3 (lower limit of normal), P10, P25, P50 (median), P75, P90, and P97 (upper limit of normal). With Microsoft Excel version 2016, trend lines were added to the centile curves of the lower limit of normal, median, and upper limit of normal, describing the equation, *R* square (*R*^2^), and standard error of the estimate (SEE) of the reference values. For cross validation, the paired samples *T* test was performed in SPSS, to demonstrate the mean difference between the reference values and the actual measured data. *p* values below 0.05 were considered as significant.

## Results

### Subject characteristics

Data from 4612 participants were eligible for analysis, involving 3671 males and 941 females. Descriptive characteristics of the participants, divided in both age categories of 10 years and sex, are given in Table 1. Females had, as expected, lower weight and height than males. The measured values of VO_2_max/kg, maximum heart rate (HR_max_), and RER are listed in Table 2, by the same division in age categories and sex.

### Interaction with age and sex

**Fig. 1:**
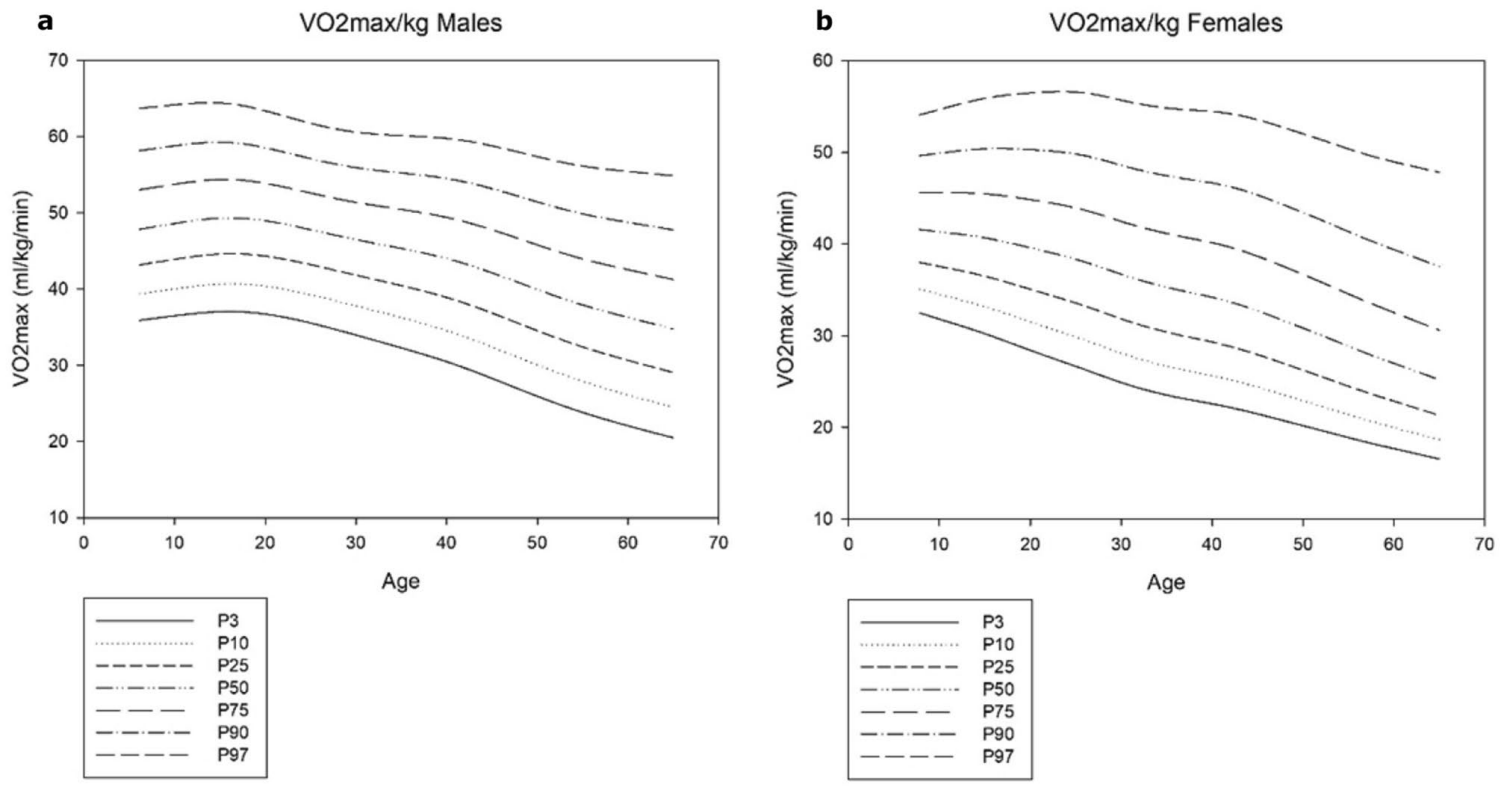
VO_2_max (ml min^−1^ kg^−1^) according to age represented as P3, P10, P25, P50, P75, P90, and P97 in **a** males and **b** females

Univariate linear regression analysis shows a negative significant effect of age on the VO_2_max/kg (*p* < 0.001) for males as well as females. Figure 1 illustrates this decline of the VO_2_max/kg with increasing age by applying centile curves. The mean value of VO_2_max/kg was 45.39 ± 8.33 ml min^−1^ kg^−1^ for males and 37.23 ± 8.71 for females. Consequently, the overall mean difference between males and females was 18.0%, ranging from 9.6% for 5–9-year old children to 23.5% for people above 60 years old.

### Reference values

Reference values for the VO_2_max/kg for both males and females were developed using the median (P50), the lower limit of normal (P3), and upper limit of normal (P97), and are depicted in Fig. 1. Table 3 displays the prediction equations with corresponding *R*^2^ and SEE values. In Table 4 the reference values are compared with several previously published values for different ages.

**Table 3:** Reference equations for the VO_2_max per kilogram

	Reference values	*R*^2^	SEE
*Males*			
Median	− 0.0049 × age^2^ + 0.0884 × age + 48.263	0.9859	1.4364
Lower limit of normal	0.0002 × age^3^ − 0.0227 × age^2^ + 0.5809 × age + 31.909	0.9996	1.4934
Upper limit of normal	0.00005 × age^3^ − 0.0065 × age^2^ + 0.0655 × age + 64.801	0.9758	0.5529
*Females*			
Median	− 0.0021 × age^2^ − 0.1407 × age + 43.066	0.9989	0.5775
Lower limit of normal	0.0017 × age^2^ − 0.3995 × age + 35.084	0.9982	0.4981
Upper limit of normal	0.0001 × age^3^ − 0.0153 × age^2^ + 0.5674 × age + 51.612	0.9894	1.3213

**Table 4:** Reference values for the VO_2_max per kilogram compared with previously published values for different ages

Reference values	Age (years)							
	8	12	20	30	40	50	60	70
*Males*								
Jones^a^	x	x	46.2	41.8	37.4	33.0	28.6	24.2
Wasserman^b^	x	x	43.3	39.6	35.8	32.1	28.4	24.7
Koch (SHIP)^c^	x	x	x	35.9	33.8	31.2	32.2	29.3
Cooper^d^	43.4	46.1	x	x	x	x	x	x
Our study	48.7	48.6	48.1	46.5	44.0	40.4	35.9	30.4
*Females*								
Jones^a^	x	x	35.8	32.2	28.6	25.0	21.4	17.8
Wasserman^b^	x	x	31.8	29.0	25.9	23.2	20.3	17.6
SHIP^c^	x	x	x	29.3	28.1	26.4	24.3	21.7
Cooper^d^	37.3	34.5	x	x	x	x	x	x
Our study	41.8	41.1	39.4	37.0	34.1	30.8	27.1	22.9

^a^Jones et al. (1985): VO_2_max/kg = 55 − 0.44 × age (years) for males and VO_2_max/kg = 43 − 0.36 × age (years) for females (Jones et al. 1985)

^b^Wasserman et al. (2005): VO_2_max = weight (kg) × [50.72 − (0.372 × age)] for males and VO_2_max = (weight + 42.8) × (22.78 − 0.17 × age) for females (Wasserman et al. 2005). For a reliable comparison, the mean weight of the subjects in our dataset was used for those ages, which are shown in Table 1

^c^Koch et al. (SHIP) (2009): for the full equation, see Koch et al. (2009) There is no comparison possible for children, because the reference values in the SHIP study are built on age categories that start at 25 years. The values of BMI (≤ 25 or > 25 kg m^−2^) were based on the mean BMI of our study population given in Table 1

^d^Cooper et al. (1984): VO_2_max = 52.8 × weight (kg) − 303.4 for boys and VO_2_max = 28.5 × weight (kg) + 288.2 for girls (Cooper et al. 1984). The weight is based on the weight of our study population, as shown in Table 1

x, reference values not suitable for this age

### Cross-validation

Supplementary data of 3135 subjects, who were not included in the primary analysis, were applied for performing cross validation. These data were derived from additional exercise tests performed at Diving Medical Center Den Helder. The sample consisted of 3017 males and 138 females. Paired samples *t* test demonstrates a mean difference of 0.03 ± 6.59 ml min^−1^ kg^−1^ with a *p* = 0.781, in favour of the predicted VO_2_max for males. For females, there is a small but significant mean difference of -1.88 ± 5.67 ml min^−1^ kg^−1^ with a *p* < 0.001, due to higher measured than predicted values.

## Discussion

The purpose of this study was to provide up-to-date reference values for the VO_2_max/kg by analysing the correlation of the VO_2_max/kg between both age and sex based on a Dutch and Flemish cohort. Data from the LowLands Fitness Registry were analysed, in which children and adults aged 6 until 65 who performed a maximal effort during exercise testing on a cycle ergometer were included.

Age showed a negative significant effect on the VO_2_max/kg (*p* < 0.001) among both sexes. Males showed higher levels of VO_2_max/kg with an overall mean difference of 18%. This difference is comparable to the literature. Using the composite equation of Cooper and Storer (Cooper and Storer 2001), the mean sex difference in VO_2_max is 16.6% at the age of 20. Our reference values were developed using the median (p50, centile curve). The lower limits of normal (p3) and upper limits of normal (p97) reference equation were established as well. These lower and upper limits are important to established normalcy of VO_2_max.

Cross-validation with data not included in the primary analysis showed no statistical difference between the predicted values and the actual measured values for males (mean difference 0.03 ± 6.59 ml min^−1^ kg^−1^, *p* = 0.781), which means that these reference values are reliable. For females, a statistical significance was found between the measured values and our reference values (− 1.88 ± 5.67 ml min^−1^ kg^−1^, *p* < 0.001), which means that our reference values predict lower values than the actual outcome. The problem might be the relatively small sample size of females available for cross validation, which is a 138 female subjects. Besides, a mean difference of 1.88 ml min^−1^ kg^−1^, although statistical significant, is small and might be therefore not of clinical relevance.

When comparing the reference values of this study to previously published values based on cycle ergometer exercise testing, it is remarkable that our reference values are higher. There are several explanations for this difference. First of all, the reference values are based on different geographic cohorts, Canadian (Jones et al. 1985), American (Wasserman et al. 2005), and German (the SHIP study, Koch et al. 2009), while our study is based on a Dutch and Flemish cohort. Physical and cultural differences between these cohorts may account for differences in the VO_2_max/kg. For instance, the VO_2_max tends to expand with increasing height, and the Dutch population is proven to be the tallest in the world (NCD Risk Factor Collaboration 2017). Besides, the VO_2_max shows a decline with increasing body mass index (BMI) (Koch et al. 2009) and mean BMI is lower in the Netherlands, compared to Canada, Germany, and the USA (26.1 compared to, respectively, 27.3, 27.4, and 29.1 kg/m^2^) (World Health Organization). In addition, since the VO_2_max is expressed in ml min^−1^ kg^−1^, a lower body weight will lead to a higher value of VO_2_max/kg. From a cultural perspective, the use of cycling in daily transfers is the most prominent in the Netherlands, compared to the rest of the world (Buehler and Pucher 2012). This may have led to an advantage in performing cycle ergometry. Furthermore, 2 of the 11 centres that submitted data to the Lowlands Fitness Registry have a military background, meaning that the physical condition of their subjects is likely to be above average, which is associated with a higher VO_2_max (Fletcher et al. 2013), and may have led to higher reference values, as well. Of interest to note is the non-linear decline in VO_2_max with age for both males and females. Traditionally, regression equations for VO_2_max are often presented as a simple linear regression of VO_2_max with age (Cooper and Storer 2001). The results of the current study do not endorse this approach. We recommend to use a non-linear approach to model VO_2_max data.

Where the studies described above only concern adults and elderly, a comparison for children is possible with the widely used data of Cooper et al. (1984). The Cooper study showed lower values, as well, but it must be noted that the difference between the Cooper study and our study is smaller than the difference between the studies discussed above and our study for boys, in particular for 12-year-old boys. However, the difference between the VO_2_max values of girls between the current study and the Cooper data are as big as the difference between adult studies. A recent case study also observed that the Cooper values for VO_2_max in children are substantially lower compared to those previously published by our group (Waterfall et al. 2020).

Taking this comparison into consideration, it is impossible to ignore the remarkable differences between our study outcome and previously published reference values, especially for adults. This demonstrates and explains the clinical experience of the existing reference values being too low for the Dutch population. Therefore, this shows the importance of having access to population-specific reference values.

Besides providing population-specific reference values, the aim of this study was to provide up-to-date values, as well. However, it is questionable whether the reference values of, for instance, Jones et al. (1985) and Wasserman et al. (2005) are outdated. Worldwide, there is a tendency towards an increase in cardiovascular diseases, of which CRF is an important marker (Ross et al. 2016). Nevertheless, this trend does not automatically mean that the health standards need to be reduced, as well. Values that relate to a standard based on desirable health conditions are officially called criterion-referenced fitness standards (Welk et al. 2011).

For people with obesity, it is impractical to use the VO_2_max/kg, since dividing the absolute value of VO_2_max (ml min^−1^) by a high body weight automatically leads to a low VO_2_max/kg. Our recommendation for people with obesity is describing the VO_2_max in absolute values (ml min^−1^) instead of relative to body mass. Then, comparison with the values of Mylius et al. (2019) is possible, who provided absolute values for the VO_2_max that were based on the LowLands Fitness Registry, as well. An alternative would be calculating a person’s ideal body weight and then dividing the absolute value of VO_2_max by this ideal weight, after which comparison with our reference values is possible.

There are multiple strengths to this study. First of all, to our knowledge, this is the first study to provide reference values for the VO_2_max/kg based on a Dutch and Flemish cohort. Second, we applied a relative substantial sample size to this study (*n* = 4612), compared to, for instance, Jones et al. (*n* = 100) (Jones et al. 1985), Wasserman et al. (*n* = 77) (Wasserman et al. 2005), and Koch et al. (*n* = 1708) (Koch et al. 2009). The different centres that submitted data to the LowLands Fitness Registry are properly distributed among the Netherlands and Flanders, and hence, the reference values are broadly representative of the Netherlands and Flanders. Besides, the data were obtained through mandatory health checks among different professions instead of voluntary health checks; consequently, there is minor selection bias at which healthier people will participate in voluntary health checks (Van de Poppe et al. 2019). Subjects with a broad age range were included, so the reference values are suitable for both children and adults from 6 until 65 years of age.

There are also some limitations to this study that need to be acknowledged. The VO_2_max/kg is the maximum amount of oxygen uptake during exercise, which is physiologically demonstrated by a plateau phase. However, during most exercise testing, the VO_2_max/kg does not reach this plateau phase. This means that the VO_2_max/kg is measured at an estimated maximum effort instead of at the physiological plateau phase. Therefore, it is possible that the definite physiological VO_2_max/kg may be a fraction higher than the estimated VO_2_max/kg used in our study. No verification procedure for VO_2_max was performed (Poole and Jones 2017). Moreover, our data were obtained by mandatory health checks among different professions, for instance divers and militaries. These professions acquire good physical condition, and therefore, the cardiorespiratory fitness might be overestimated, compared to the general Dutch population. Furthermore, our database contained almost 4 times as many male subjects as female subjects, and hence, the accuracy of our reference values might be higher for males than females. Finally, there is a skew distribution of subjects among the different age categories, since the decades of 20 s through the 50 s are most represented.

To provide more reliable reference values for females, future research with more female subjects is needed. Another recommendation is including more subjects in the lowest and highest age categories, especially subjects of 4 until 10 years old and above 65 years, to provide reference values with more reliability for children and elderly, as well.

## Conclusions

This study provided reference values for the VO_2_max/kg using cycle ergometry as mode for CPET based on both age and sex, and showed reliable results during cross-validation testing, especially for males. Comparison demonstrated remarkable higher levels of VO_2_max/kg using the reference values of our study than those of previous published studies. Therefore, our reference values should be able to result in more accurate interpretations of measured VO_2_max/kg for specifically the West-European population.

## Abbreviations

BMI: Body mass index
CPET: Cardiopulmonary exercise testing
CRF: Cardiorespiratory fitness
CVD: Cardiovascular diseases
HR_max_: Maximum heart rate
RER: Respiratory exchange ratio
VO_2_max: Maximum oxygen uptake
